# High Frequency Audiometric Study in Cancer-Cured Patients Treated with Cisplatin

**DOI:** 10.1016/S1808-8694(15)30572-3

**Published:** 2015-10-19

**Authors:** Elizabeth Oliveira Crepaldi de Almeida, Waléria Gama Umeoka, Rafaela Corcelli Viera, Ilmara Fátima de Moraes

**Affiliations:** 1 Doctoral degree in teaching, full professor in the Pontifícia Universidade Católica de Campinas (Campinas Pontificial Catholic University).; 2 Graduated in speech therapy; speech therapist.; 3 Graduated in speech therapy; speech therapist.; 4 Master's degree in psychology, presiding researcher of the Associação Beneficente São Lucas (Sao Lucas Beneficent Association). Pontifícia Universidade Católica de Campinas (Campinas Pontificial Catholic University).

**Keywords:** antineoplastic drugs, audiometry, cancer, ototoxicity

## Abstract

Hearing loss has been described in patients undergoing chemotherapy, given the ototoxic nature of these drugs. An audiological investigation is relevant in such cases.

**Aim:**

to assess audibility thresholds at high frequencies in individuals with cancer that was treated successfully with cisplatin and its associations, to verify possible hearing loss as a side effect of therapy. Site and date of the study: Campinas, Sao Paulo, in 2006.

**Material and Method:**

Ten volunteers aged between 5 and 27 years were assessed by a clinical history, otoscopy, and conventional and high frequencies audiometry in this clinical and experimental study.

**Results:**

A kappa coefficient statistical analysis revealed significant differences between ears in 50% of 14 frequencies that were evaluated. Eight participants presented hearing losses, which started at 1 kHz, increasing markedly at 6 kHz and above. Fisher's Exact Test revealed a significant association only with the dose and the right ear at high frequencies.

**Conclusion:**

It is possible that the hearing loss detected in this study is at least partially due to the ototoxicity of antineoplastic drugs; such loss may occur even after treatment is interrupted. We suggest that a protocol for audiological follow-up of patients undergoing chemotherapy should be created.

## INTRODUCTION

Cisplatin is a potent anticancer drug that is widely used in the treatment of advanced cancer in adult and children. One of its side effects is ototoxicity, which related directly with the dose and the administration route; a single high dose may affect hearing more intensely than the same amount given in fractionated doses.^1^, ^2^ Cisplatin is used against various cancer types in humans, such as testicle and ovarian cancer, head and neck cancer, lung cancer and germinative cell cancer. This drug is effective in chemotherapy when given by the intraperitoneal or endovenous routes, and has a therapeutic potential in many malignancies, particularly those mentioned above.^3^ Given a certain blood concentration, toxic substances may reach the organ of Corti and the sensorineural epithelia of the posterior labyrinth by means of the labyrinthic fluids. Ototoxic drugs result in cochlear and/or vestibular symptoms. These symptoms may progress slowly and insidiously, even after the drug has been withdrawn; there is generally a direct relation between the dose and the severity of otological injury. The longer a toxic substance remains in the organism, the more severe will its harmful effect be, although there may be individual differences in the response to such agents, as well as the part played by a family history of hearing loss, noise sensitivity, among others.^4^

Individuals using chemotherapy, for instance with cisplatin, could avoid degeneration of the organ of Corti if ototoxicity were monitored and/or detected early.^5^ Some authors argue that high frequency pure tone audiometry helps detect ototoxicity due to cisplatin, and that frequencies of 12,000 Hz and 14,000 Hz are especially important. Hearing data may be altered by changes in the dosage of drugs or in treatment methods.^1^ High frequency pure tone audiometry investigates cochlear baseline responses, assessing audition from 8,000 to 20,000 Hz, depending on the clinical audiometer that is used.^6^, ^7^ It is currently considered as a relevant tool for the early diagnosis of sensorineural hearing loss, which usually begins at higher frequencies.6-8 Investigation of patients undergoing clinical therapy with ototoxic drugs has underlined the important role of high frequency audiometry in monitoring hearing loss.^1^, ^5^, ^9^

Further studies have shown that high frequencies are needed for discriminating between consonants and for recognizing speech; individuals with hearing loss of any degree at these frequencies find it difficult to understand speech in noisy environments.^10^

The main use of high frequency audiometry is in monitoring the audition of patients in which hearing losses are suspected; the main conditions are ototoxicity, sequelae of otitis media, chronic renal failure, presbyacusis, assessment of auditory processing, investigation of auditory changes in families with inherited hearing loss, and monitoring of individuals that are frequently exposed to noise.^11^, ^12^

### Cancer

Cancer is a range of disease in various sites and of different morphological types, which have in common two main biological features: uncontrolled cell growth and the ability to extend beyond the tissue of origin. Variability exists relative to the stage in which each cancer is detected by available diagnostic methods.^13^

Germ cell tumors, also known as teratomas, endodermal sinus tumors or embryogenic carcinomas, originate from the aforementioned germ cells, and follow an abnormal path. When cells in these tumors begin to divide, tumors are formed with parts of hair, teeth, skin and others. In different cases, germ cells originate malignancies, behaving aggressively. Such germ cell tumors may occur at any age in childhood; testicles, ovaries, the vagina, the sacrum and coccyx (lower vertebrae) are frequently involved, as well as less frequent sites such as the head and neck, the liver and the brain. These tumors, however, are less frequent compared to leukemia and brain tumors.^14^

Neuroblastomas are about 8% of cancers in children aged below 15 years. This is a solid tumor that develops in nervous tissues located in the neck, the thorax, the abdomen, the pelvis or in adrenal glands. About 97% of neuroblastomas are sympathetic nervous system embryogenic malignancies, occurring almost exclusively in the newborn and young infants. Unlike germ cell tumors, neuroblastomas are the most common cancers in the first years of life; its incidence is twice that of leukemia. In many cases when it is diagnosed, it has already metastasized to the lymphnodes, liver, lungs, bone and bone marrow. The neuroblastoma is primarily a tumor of young children. Two thirds of neuroblastoma cases are diagnosed in children aged below 5 years.^15^

Another tumor type is the rabdomyossarcoma, a malignancy that originates in primitive mesenchymal cells, at any site of the body. It is the most common soft tissue sarcoma in infancy, and is usually located in the head and neck.^16^

The osteosarcoma is a primary bone malignancy that occurs in children, teenagers and young adults. Its peak incidence is in the second decade of life; it is about 5% of the malignancies in childhood and adolescence.^17^

Cisplatin ototoxicity and high frequency pure tone audiometry

High frequency audiometry has been suggested as a method for monitoring the effect of ototoxic drugs on hearing.^18^, ^19^, ^20^ There are many ototoxic substances - other than cisplatin - used for treating a variety of cancers, such as carboplatin, actinomycin, bleomycine, nitrogen mustard compounds (ex: mustine), misonidazole, vincristine and vinblastine.^4^, ^9^ A study using conventional and high frequency audiometry assessed 62 individuals treated with cisplatin; pre and post testing were done in a time-series (each three weeks during therapy and across three months post-therapy). Results indicated hearing loss mainly at high frequencies due to cisplatin.^21^ Another study monitored cisplatin-treated cancer patients with conventional (250 to 8,000 Hz) and high frequency audiometry (9,000 to 20,000 Hz). All patients had normal hearing before cancer therapy. Testing was done at each phase of chemotherapy. There were point and progressive high frequency alterations; after one or two chemotherapy sessions, 100% of patients had hearing loss at and above 9,000 Hz. These patients also complained of tinnitus and difficulties in understanding speech in the presence of background noise.^22^ This study assesses the possible side effects of chemotherapy on the hearing of successfully treated cancer patients, considering the ototoxic effects of cisplatin and other anticancer drugs. The sample included subjects that had cancer that was treated with cisplatin and associated drugs during variable periods; these subjects had been discharged a few years before this study was undertaken. The current study is an uncontrolled post-test assessment.

## PURPOSE

To assess high frequency auditory thresholds in successfully treated cancer patients aged from 5 to 27 years, treated with cisplatin and associated drugs, to verify possible hearing loss as a sequel of therapy.

## SPECIFIC OBJECTIVES

To assess, in patients treated with cisplatin and associations, possible differences in the ototoxic effect between the ears that were evaluated, the incidence of auditory changes, the number of affected ears at each frequency, the comparison between conventional and high frequency audiometry, and the historic variables that might have influenced the hearing of these patients.

## MATERIAL AND METHOD

Participants The sample included volunteers who visited a speech therapy clinic located in the city of Campinas (Sao Paulo state) after being contacted by telephone from the hospital in which they had been treated for cancer. Fifty individuals were contacted, of which 10 accepted the invitation to participate in this study.

These 10 subjects, of both genders, were aged between 5 and 27 years at the time data were collected, and had started cancer therapy at ages 6 months to 13 years. All had been cured at the time this study was done. All had been treated with cisplatin, which is ototoxic,1,2,4,5,9 associated with other drugs, some of which were also ototoxic. An otorhinolaryngologist assessed all participants before the research audiological tests were done, as part of the method. Other drugs that the subjects had used in cancer therapy included doxorubicin, vincristine, actinomycin D, cyclophosphamide and etoposide. Of these, vincristine and actinomycin D are also ototoxic.^4^ The attending oncologist at the hospital in which the participants had been treated, all patients underwent a standard medication protocol consisting of giving the drugs jointly in doses that depend on the type and extension of the tumor, as well as the height and weight of the patients. Thus, all patients had been exposed to more than one ototoxic substance simultaneously. Participants were characterized according to a protocol at the time the clinical history was taken. These data are presented on Table 1 (the variable “dose” refers to the use of cisplatin and associated drugs).

**Table 1:** Participant data.

Participant	Current age	Gender	Type of tumor	Type of treatment	Age at which treatment was started	Dose	Duration of therapy	Number of cycles	Audiological monitoring prior to study *
1	5	F	Osteossarcoma	Chemotherapy	10 months	780 mg/m²	10 months	2 cycles each week and 3 cycles each 4 weeks	No
2	19	F	Neuroblastoma with metastasis	Chemotherapy	13 years	480 mg/m²	2 years	4 cycles each 6 weeks	Yes, the year before; diagnosed with moderate to severe sensorineural dysacusis
3	8	M	Abdominal neuroblastom	Chemotherapy	6 months	200 mg/m²	6 months	2 cycles each 9 weeks	No
4	15	F	Rabdomyossarcoma below the bladder	Chemotherapy	2 years	400 mg/m2	2 years	4 cycles each 21 days	No
5	10	F	Abdominal germinative tumor - yolk sac	Chemotherapy	1 year	300 mg/m²	4 months	3 cycles each 3 weeks	Yes. Used hearing aid 4 years.
6	27	M	Osteossarcoma	Chemotherapy	13 years	400 mg/ m²	4 months	4 cycles each 6 weeks	Yes, two months before as part of admission test..
7	11	M	Abdominal neuroblastoma	Chemotherapy	1 year	200 mg/m²	1 year	year cycles each 9 weeks	No
8	20	F	Rabdomyossarcoma	Chemotherapy	2 years	360 mg/m2	2 years	4 cycles each 3 weeks	No
9	22	M	Carcinoma of rhinopharynx	Chemotherapy	12 years	480 mg/ m²	2 years	4 cycles each 3 weeks	Yes, as part of admission test
10	19	F	Carcinoma of rhinopharynx	Chemotherapy + Radiotherapy	8 years	480 mg/m²	2 years	4 cycles each 3 weeks	Yes, twice (2004 and 2005), no change

* Data provided by participants or caretakers.

Table 1 shows that the time period between chemotherapy and the audiological testing done in this study varied from 3 and a half years and 16 years.

The following was noted about the otological history: participant 1 reported otitis soon after chemotherapy; participant 2 complained of tinnitus, dizziness, hearing loss and headaches, but did not report when these symptoms started; participant 5 reported bilateral pruritus and hearing loss; participant 6 reported tinnitus and pruritus; participant 7 reported hearing loss; participant 8 reported tinnitus and otalgia; participant 10 reported otalgia. Participants 3, 4 and 9 had no otological complaints.

### Material

A clinical history script for obtaining data about participants was written. The following equipment was used in audiological testing: a TK otoscope for inspecting the external auditory canal. An Interacoustics AC40 Clinical Audiometer with two frequency fields, one at 128 to 8,000 Hz and the other at 8,000 to 20,000 Hz, according to the ANSI S3.6 (1989) and ISO 389 standards. Telephonics TDH 39 P headphones were used for conventional audiometry (250 to 8,000 Hz); Koss R/80 headphones were used for high frequency audiometry (9,000, 10,000, 11,200, 12,500, 14,000 e 16,000 Hz). An acoustic booth was used. The SAS - system for Windows - V8, was used for the analysis of results. Procedure The Research Ethics Committee approved the project and the free informed consent form (Protocol 620/05), after which participants were informed about the testing procedures to clarify any doubts. Patients that agreed to participate signed the free informed consent form. The following procedures were applied to all patients:

a) Clinical history taking;
b) Inspection of the external ear to verify the presence of ear wax and/or foreign bodies or secretion in the external auditory canal that might interfere with testing;
c) Conventional pure tone audiometry and high frequency audiometry, done in an acoustic booth.

The descending technique was used for obtaining auditory thresholds at 250, 500, 1,000, 2,000, 3,000, 4,000, 6,000, 8,000, 9,000, 10,000, 11,200, 12,500, 14,000 and 16,000 Hz (conventional audiometry e high frequency audiometry). Threshold testing was started by reducing the intensity of the sound stimulus in 10 dB intervals until no further response was obtained. Next, the stimulus was presented 5 dB above that level and then decreased again in 5 dB intervals until no further response was obtained. The auditory threshold was considered as the lowest intensity at which a response was obtained.

The procedure was applied to both ears of each patient in a single session lasting about 20 minutes. Data were treated statistically using different methods, according to the aims of this study.

Criteria for establishing the presence of auditory alterations In this study, any threshold change higher than 25 dBNA in adults and 15 dBNA in children^23^ for conventional pure tone and high frequency audiometry confirmed the presence of auditory alterations.

## RESULTS

### Comparison between ears

The kappa (k) statistics index was used to check possible differences in ototoxicity between ears. This statistical test is a measure of agreement used in nominal scales for verifying by what amount observations differ from expected variations by chance. K statistical values range from 0 to 1, where 0 indicates lack of agreement beyond chance and 1 is perfect agreement.^24^ In this study, agreement between both ears was sought, as the intention was to assess if measurements were similar regardless of the tested ear. The interpretation of results is done according to k values where 0 indicates poor agreement, over 0 to 0.20 is slight agreement, 0.21 to 0.40 is moderate agreement, 0.41 to 0.60 is considerable agreement, 0.61 to 0.80 is substantial agreement, and 0.80 to 1 is excellent agreement. Furthermore, the standard error in K statistics makes it possible to estimate its statistical significance and its 95% confidence interval.24 In this study, a weighted kappa coefficient below 0,75 was considered as the criterion for defining the presence of a difference between ears. Values closer to 1 indicated that there was more evidence showing that both ears responded similarly to stimuli. The confidence interval was 95%, but the exact estimated value was between the maximum and minimum interval value.^24^

In this study, close frequencies (for example, 8KHz and 9KHz) were calculated as different but with less weight in weighted k statistics. The weighted k value was considered as evidence of a larger difference between frequency categories that are more distant from each other. See Table 2. There was a significant difference between ears in 50% of the 14 frequencies that were assessed, according to the weighted kappa; there was no statistically significant difference in the other 50% of frequencies.

**Table 2:** Comparison between right and left ears for each tone threshold (in dBNA) that was assessed, as a function of kappa statistics (n=20).

Frequency	RE Means	LE Means	Kappa	Weighted kappa	Result
0,25 kHz	14	8	01,14	0,47	Different
0,5 kHz	11,5	6	0,11	0,36	Different
1 kHz	14	12	0,41	0,76	No Difference
2 kHz	14,5	11	0,37	0,66	Different
3 kHz	16,5	16,5	0,25	0,79	No Difference
4 kHz	26,5	19	0,42	0,56	Different
6 kHz	49,5	45,5	0,33	0,80	No Difference
8 kHz	47,5	46	0,39	0,78	No Difference
9 kHz	56	56,5	0,38	0,75	No Difference
10 kHz	57	59,5	0,31	0,68	Different
11,2 kHz	56	56	0,29	0,78	No Difference
12,5 kHz	58,5	57,5	0,28	0,74	Different
14 kHz	49,5	49,5	0,04	0,70	Different
16 kHz	45	53	0,51	0,85	No Difference

Incidence of auditory alterations Eight of 10 participants assessed by conventional pure tone audiometry and high frequency audiometry presented hearing loss, while two had hearing within normal limits for their age, according to the criteria described under Method. Table 3 shows the distribution of cases.

**Table 3:** Audiometry results in 10 patients cured of cancer, having used cisplatin chemotherapy.

PARTICIPANT	RESULTS OF AUDIOMETRY
1	Bilateral symmetrical hearing loss at and above 6,000 Hz
2	Bilateral symmetrical hearing loss at and above 6,000 Hz
3	No hearing loss
4	Non-symmetrical bilateral hearing loss - RE at and above 1,000Hz; LE at and above 10,000 Hz
5	Bilateral symmetric hearing loss at and above 1,000 Hz
6	Bilateral symmetric hearing loss at and above 4,000 Hz
7	Unilateral left hearing loss between 9,000 and 11,200 Hz
8	Bilateral symmetric hearing loss at and above 6,000 Hz
9	No hearing loss
10	Bilateral symmetric hearing loss at and above 6,000 Hz

Auditory alterations relative to the affected frequencies

**Figure 1:**
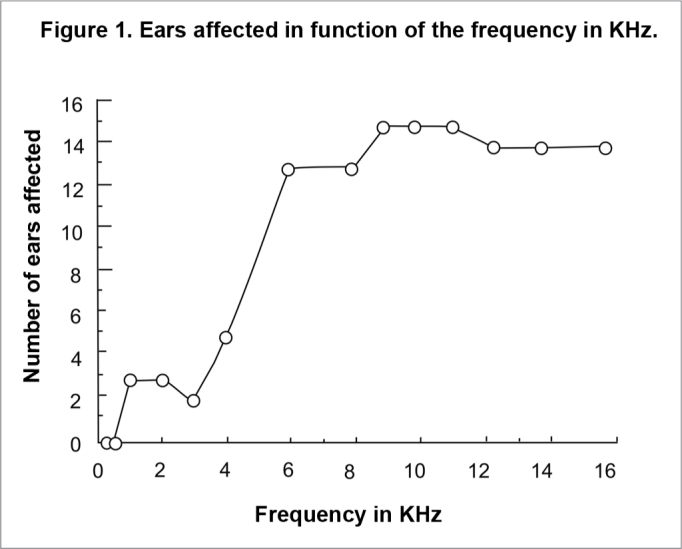
Cumulative curve of affected ears as a function of auditory frequency, in kHz.

Table 4 and Figure 1 show that in this sample of patients treated with ototoxic drugs, including cisplatin, ear involvement occurred at and above 1 kHz, and that there was marked involvement at and above 6kHz.

**Table 4:** Number of affected ears by auditory frequency

Frequency	Ears
0,25 kHz	0
0,5 kHz	0
1 kHz	3
2 kHz	3
3 kHz	2
4 kHz	5
6 kHz	13
8 kHz	13
9 kHz	15
10 kHz	15
11,2 kHz	15
12,5 kHz	14
14 kHz	14
16 kHz	14

Comparison between the incidence of auditory alterations in conventional audiometry and high frequency audiometry.

Table 4 and Figure 1 shows that there is a clear difference in two sets of data; there is a small group of ears that were affected at frequencies up to 4,000 Hz, and a larger group of ears that were affected at and above 4,000 Hz.

Clinical historic variables that altered the hearing of patients

The literature states that cisplatin ototoxicity is directly related to the dose and the manner by which the drug is given.^1^, ^2^, ^4^ These aspects, therefore, were investigated using Fisher's exact test according to the following variables: age at which chemotherapy was started, the duration of therapy, the dose of cisplatin and associated drugs, and audiometric results. Fisher's exact test calculates the probability of association or independence between the variables that are being analyzed; it establishes the probability of an association between variables being due to chance. If statistical significance is p ≤ 0.05 in this test, then the association between variables is not due to chance.^25^ Findings were treated separately for the right and left ears and for conventional pure tone audiometry and high frequency audiometry, as shown on Tables 5, 6, 7 and 8.

**Table 5:** Assessment of the mean duration of chemotherapy, the dose and the age (beginning of therapy) for the groups with and with no right ear alterations at conventional frequencies

	Mean with no changes	Mean with alterations	P	Result
Duration of chemotherapya	14 meses	16,2 meses	0,417	No difference
Dose (mg/m²)	560	457,1	0,167	No difference
Age (months)	54	68,28	0,550	No difference

**Table 6:** Assessment of the mean duration of chemotherapy, the dose and the age (beginning of therapy) for the groups with and with no left ear alterations at conventional frequencies

	Mean with no changes	Mean with alterations	P	Result
Duration of chemotherapya	16,5 months	15 months	0,714	No difference
Dose (mg/m²)	320	466,6	0,330	No difference
Age (months)	46,5	75,6	0,186	No difference

**Table 7:** Assessment of the mean duration of chemotherapy, the dose and the age (beginning of therapy) for the groups with and with no right ear alterations at high frequencies

	Mean with no changes	Mean with alterations	P	Result
Duration of chemotherapya	9 months	17,25 months	0,089	No difference
Dose (mg/m²)	200	460	0,044*	Different
Age (months)	9	77,7	0,800	No difference

* p < 0,05

**Table 8:** Assessment of the mean duration of chemotherapy, the dose and the age (beginning of therapy) for the groups with and with no left ear alterations at high frequencies-

	Mean with no changes	Mean with alterations	P	Result
Duration of chemotherapya	6 months	16,6 months	0,50	No difference
Dose (mg/m²)	200	431,1	0,50	No difference
Age (months)	6	71,1	1,00	No difference

Fisher's exact test revealed a significant association only for the variable dose and the right ear at high frequencies. There was no association between variables in the remaining conditions.

## DISCUSSION

Concerning a possible difference in ototoxic action between ears, the kappa statistics suggested differences between the right and left ears in half of the sample. As to the incidence of auditory alterations in the sample, eight participants had hearing loss. The data revealed that there was significant hearing loss at around 6,000 Hz in 5 cases, suggesting that conventional audiometry would be sufficient to diagnose high frequency involvement. Apparently in these cases high frequency audiometry would have no preventive value, as conventional audiometry would provide enough data for justifying preventive measures, such as recommending that the patient undergo otorhinolaryngological and phonoaudiological monitoring due to the possibility of progression of hearing loss. Participant 5 had already started using a hearing aid for four years; in this case chemotherapy had ended 3 and a half years before starting to use the hearing aid. Hearing loss was present at and above 1,000 Hz according to the audiometric evaluation; also in this case, high frequency would have had no preventive value.

The results of participant 4 deserve further analysis. In this case, there was bilateral non-symmetrical hearing loss (RE at and above 1,000 Hz and LE at and above 10,000Hz). Conventional pure tone audiometry (up to 8,000 Hz) had shown 10 to 15 dBNA in the left ear at all frequencies, in other words no loss. High frequency audiometry of this ear, however, showed progressive loss; it was 35 dBNA at 10,000Hz and showed no response at 14000 and 16000Hz. In this case, high frequency audiometry made it possible to identify the onset of a degenerative process, which would not have been perceived in conventional audiometry. In this case, high frequency audiometry was effective in detecting a problem, allowing preventive monitoring and interventions.

Participant 7 also stood out; there was unilateral left hearing loss between 9,000 and 11,200 Hz. Conventional audiometry was within normal limits. High frequency audiometry made it possible to detect the frequency range in which the results were from 20 to 30 dbNA. This was the most significant case of the sample, since it was possible to detect the onset of high frequency auditory degeneration, even though these changes were small from the standpoint of auditory acuity. In this case, high frequency audiometry demonstrates its preventive value; the audiogram suggests a trend towards worsening of hearing loss, particularly when considering the patient's age (11 years). Audiological information, thus, makes it possible to recommend monitoring and possible interventions. Analysis of hearing loss at each frequency shows that ears were affected at and above 1 kHz, and that there was a marked increase in hearing loss at and above 6 kHz. This suggests that in this sample, high frequency audiometry could have identified hearing losses in all 8 participants with hearing loss, even if it had been the only test. Confirmation of this is seen in the comparison between conventional pure tone and high frequency audiometry as applied to this sample. Two data sets were clearly identified; one composed of ears with losses up to 4,000 Hz and another composed of ears with losses at and above this frequency. Thus, contrary to what might have been suggested above, high frequency audiometry may be considered an important tool for detecting and monitoring hearing losses, which is in agreement with the literature.^18^, ^19^, ^20^

Of all the variables affecting the hearing of patients, such as right and left ears, the presence or absence of hearing loss, the mean age at which chemotherapy was started, the duration of therapy, drug dosages, conventional and high frequency audiometry, only the variable drug dose (mg/m2) in the right ear at high frequencies was significantly different (P=0.044; p<0.05). This points, therefore, to evidence suggesting that the chemotherapy drug dosage used by the participants included in our sample may have had an important role in causing hearing loss, notwithstanding the time elapsed since the end of chemotherapy and this study and the impossibility at this point of establishing its effect isolatedly. These results are consistent with the literature that was presented at the beginning.^1^, ^2^, ^4^, ^5^, ^9^, ^18^, ^19^, ^20^

## CONCLUSION

This study aimed to assess high frequency auditory thresholds in subjects aged 5 to 27 years that had been successfully treated for cancer with cisplatin and associated drugs to test for possible hearing loss as a sequel of therapy. Although the variable drug dose was significantly different only for the right ear at high frequencies, it is possible that hearing loss detected in 8 of 10 participants is due, at least in part, to the ototoxic effect of anticancer drugs (cisplatin + vincristine + actinomycin D + associated drugs). Such hearing loss may occur slowly and insidiously, especially because of the drug dose and the duration of these substances in the body, even after treatment has ceased.4 This study points to the importance of introducing routine audiological monitoring in chemotherapy for cancer patients.
